# Cancer Cells Resistant to Therapy Promote Cell Surface Relocalization of GRP78 Which Complexes with PI3K and Enhances PI(3,4,5)P3 Production

**DOI:** 10.1371/journal.pone.0080071

**Published:** 2013-11-11

**Authors:** Yi Zhang, Chun-Chih Tseng, Yuan-Li Tsai, Xiaoyong Fu, Rachel Schiff, Amy S. Lee

**Affiliations:** 1 Department of Biochemistry and Molecular Biology, University of Southern California, Keck School of Medicine, USC Norris Comprehensive Cancer Center, Los Angeles, California, United States of America; 2 Breast Center, Baylor College of Medicine, Houston, Texas, United States of America; Duke University Medical Center, United States of America

## Abstract

Traditionally, GRP78 has been regarded as an endoplasmic reticulum (ER) lumenal protein due to its carboxyl KDEL retention motif. Recently, a subfraction of GRP78 is found to localize to the surface of specific cell types, serving as co-receptors and regulating signaling. However, the physiological relevance of cell surface GRP78 (sGRP78) expression in cancer and its functional interactions at the cell surface are just emerging. In this report, we combined biochemical, imaging and mutational approaches to address these issues. For detection of sGRP78, we utilized a mouse monoclonal antibody highly potent and specific for GRP78 or epitope-tagged GRP78, coupled with imaging and biochemical techniques that allowed detection of sGRP78 but not intracellular GRP78. Our studies revealed that breast and prostate cancer cells resistant to hormonal therapy actively promote GRP78 to the cell surface, which can be further elevated by a variety of ER stress-inducing conditions. We showed that sGRP78 forms complex with PI3K, and overexpression of sGRP78 promotes PIP3 formation, indicative of PI3K activation. We further discovered that an insertion mutant of GRP78 at its N-terminus domain, while retaining stable expression and the ability to translocate to the cell surface as the wild-type protein, exhibited reduced complex formation with p85 and production of PIP3. Thus, our studies provide a mechanistic explanation for the regulation of the PI3K/AKT signaling by sGRP78. Our findings suggest that targeting sGRP78 may suppress therapeutic resistance in cancer cells and offer a novel strategy to suppress PI3K activity.

## Introduction

The 78 kDa glucose-regulated protein (GRP78), also referred to as BiP/HSPA5, is a major endoplasmic reticulum (ER) chaperone with anti-apoptotic properties [1] and a master regulator of ER stress signaling [2], [3]. Tumor cells are subjected to ER stress due to intrinsic factors of altered metabolism and extrinsic factors of hypoxia and nutrient deprivation. ER stress induction of GRP78 in cancer cells favors cell survival, tumor progression [4], [5] and confers drug resistance in both proliferating and dormant cancer cells, as well as tumor associated endothelial cells [6]–[11]. Therefore, understanding how GRP78 exerts its pleiotrophic effects on cell proliferation and survival is of major importance.

Traditionally GRP78 has been regarded as an ER lumenal protein due to its carboxyl KDEL retention motif [12]. Recently, a subfraction of GRP78 was found to localize to the surface of specific cell types, particularly in cancer cells [13]–[16]. Cell surface proteome profiling of tumor cells revealed a relative abundance of heat shock chaperones and glucose-regulated proteins, including GRP78 [17]. Importantly, preferential expression of GRP78 on the surface of tumor cells but not in normal organs enables specific tumor targeting, leading to tumor suppression without harmful effects on normal tissues [18]–[21].

Evidence is emerging that sGRP78 can form complexes with specific cell surface proteins and regulate signal transduction [13], [14], [16], such as being a co-receptor for the proteinase inhibitor α2-macroglobulin (α2-M*) induced signal transduction for cancer survival and metastasis [22], [23]. Cripto, a GPI-anchored cell surface protein key to human tumor progression, and sGRP78 form a complex and collaborate to inhibit TGF-β signaling and enhance cell growth and PI3K/AKT activation [24], [25]. Additionally, sGRP78 is required for T-cadherin-dependent endothelial cell survival [26], activation of apoptosis mediated by Kringle 5 [27], [28] and extracellular Par-4 and TRAIL [29], as well as viral entry into host cells [30], [31]. Recently we demonstrated cell surface localization of GRP78 is regulated by ER retrieval machinery and enhanced by depletion of Ca^2+^ from the ER [32]. Cancer cells are often subjected to ER stress, which are aggravated by cytotoxic therapy leading to resistance. However, whether pathological stress, such as development of therapeutic resistance, leads to relocalization of GRP78 to the cell surface is not known.

The PI3K/AKT pathway is activated in a wide array of cancers leading to proliferation and therapeutic resistance [33]. The PI3K has two subunits, the p85 regulatory subunit and the p110 catalytic subunit. For PI3K activation, tyrosine phosphorylation of the p85 regulatory subunit of PI3K relieves its inhibitory activity on PI3K, leading to its activation. Upon binding to the activated growth factor receptor, PI3K is recruited to the plasma membrane. PI(4,5)P2 is phosphorylated by PI3K to yield PI(3,4,5)P3, which promotes membrane localization of PDK1, which then phosphorylates and activates AKT. Through knockdown of GRP78 by siRNA, ligation of cell surface GRP78 with antibody and in genetic models of cancer, GRP78 has been established as a novel regulator of PI3K signaling both in vitro and in vivo [16], [25], [34], [35]. While there can be multiple mechanisms whereby GRP78 can affect AKT activation, it has been reported that antibody targeting the N-terminus of GRP78 mimics the receptor-recognized forms of α2-M* as a ligand and drives PI3K-dependent activation of AKT and subsequent stimulation of cellular proliferation in vitro [21], [36]. Conversely, a carboxyl terminal domain reactive antibody acts as an antagonist of α2-M* and suppresses α2-M*-induced AKT phosphorylation [21]. Recently, a monoclonal antibody targeting cell surface GRP78 is shown to suppress PI3K/AKT signaling, tumor development and metastasis in multiple cancer models [37]. Despite these advances, little is known on how sGRP78 regulates PI3K activity. In this report, we analyzed sGRP78 expression in breast and prostate cancer cell lines resistant to hormonal therapy, and examined its regulation of PIP3 production. These results expand our knowledge on sGRP78 and have important implications for cancer therapy.

## Materials and Methods

All protocols for animal use were reviewed and approved by the USC Institutional Animal Care and Use Committee. The animal assurance number is A3518-01. The protocol number is 9964.

### Cell lines and culture

Mouse embryonic fibroblast (MEF) cells [38], human cell lines, HEK293T [32] and HeLa [32], were cultured in Dulbecco's modified Eagle medium (DMEM) containing 10% fetal bovine serum (FBS) and 1% penicillin/streptomycin. Human cell lines, LNCaP (ATCC, Manassas, VA) and C4-2B (Viromed Lab, Minneapolis, MN), were maintained in RPMI 1640 medium supplemented with 10% FBS and 1% penicillin/streptomycin. The MCF7L parental cells [39] were cultured in RPMI 1640 supplemented with 10% heat-inactivated FBS and 1% penicillin/streptomycin/glutamine; the MCF7L-TamR cells were in the same medium except for the phenol red free (PRF) RPMI 1640 and 10% charcoal dextran stripped (CS)-FBS. The parental MCF7/HER2-18 cells [40] were cultured in DMEM containing 10% FBS, 1% penicillin/streptomycin, 0.4% geneticin (Life Technologies, Grand Island, NY) and 15 µg/ml insulin (Sigma-Aldrich, St. Louis, MO); the MCF7/HER2-18-TamR cells were in the same medium except for the PRF DMEM and 10% CS-FBS. The TamR cells of both MCF7L and MCF7-HER2-18 models were maintained in medium containing 100 nM 4-hydroxytamoxifen (Sigma-Aldrich) as the final concentration. The estrogen-dependent cell line, MCF-7/BUS, was kindly provided by A.M. Soto (Tufts University, Medford, MA) and has been described [41], [42]. The isolation and culture conditions for the estrogen starvation resistant clone MCF-7/BUS-10 have been described [43]. All cells were maintained at 37°C in a humidified atmosphere of 5% CO_2_, 95% air. For stress treatment, the cells were treated with thapsigargin (Tg) at 300 nM, tunicamycin (Tu) at 1.5 µg/ml for 16 h, or 2-deoxyglucose (2DG) at 10 mM for 24 h.

### Plasmids

The construction of FLAG-GRP78 with a FLAG-tag inserted after the ER signal peptide (aa 1–18) of full length human GRP78 (aa 1-654) has been described [32]. GRP78-103F containing a FLAG-tag inserted right after aa 103 of human GRP78 was constructed as follows: full-length human GRP78 in pcDNA3 (Life Technologies) backbone was used as template with QuikChange site-directed mutagenesis (Agilent Technologies, Santa Clara, CA). The full length human PI3K regulatory subunit, p85 alpha cDNA, was amplified by RT-PCR from human HEK293 RNA and subcloned into pcDNA3 at BamHI and XbaI sites.

### Transfection conditions

The cells were cultured to 60–80% confluence and transfected with BioT (Bioland Scientific, Paramount, CA) following the manufacturer's instructions as described [32].

### Immunoblot analysis

Cells were lysed in radioimmunoprecipitation (RIPA) buffer supplemented with competent protease inhibitor (Thermo Scientific, Rockford, IL). The cell lysates were subjected to 10% SDS gels and Western blot analyses as described [32]. Antibodies used were: mouse anti-FLAG antibody (Sigma-Aldrich), 1∶1000; mouse anti-GRP78 antibody MAb159 (gift from P. Gill, USC), 1∶2000; rabbit anti-PI3K p85 antibody (#4292, Cell Signaling Technology, Danvers, MA), 1∶1000; rabbit anti-PI3K p110α antibody (#4249, Cell Signaling Technology, Danvers, MA), 1∶1000; mouse anti-EphB4 antibody (gift from P. Gill, USC), 1∶1000; rabbit anti-NKA α1 (Na, K-ATPase α1) antibody (Cell Signaling Technology), 1∶1000; mouse anti-β-actin antibody (Sigma-Aldrich), 1∶5000. Experiments were repeated 2–3 times. Protein levels were detected either by enhanced chemiluminescence (ECL) or fluorescence staining, and visualized and quantitated with Image Lab (Bio-Rad Laboratories, Hercules, CA) or by LiCor Odyssey.

### Immunofluorescence and confocal microscopy

For detection of endogenous sGRP78, C4-2B cells were seeded at 5×10^3^ per well in an 8-well chamber slide (Millipore, Billerica, MA) for 24 h and then treated with Tg for 8 h. The cells were then fixed in cold 4% paraformaldehyde for 10 min, washed with cold PBS, and then blocked with 5% BSA in PBS (BSA/PBS) on ice for 30 min. The cells were stained with mouse anti-GRP78 monoclonal antibody (MAb159) in 1% BSA/PBS at 10 µg/ml at 4°C overnight without permeabilization. The cells were washed with cold PBS and stained with AlexaFluor-488 goat anti-mouse antibody (Life Technologies) in 1% BSA/PBS at 4°C for 1 h. For subsequent immunostaining for p85 which is intracellular, the cells were permeabilized with 0.5% (w/v) saponin in PBS at room temperature (RT) for 15 min. The cells were washed with PBS containing 0.01% saponin (PBS/Sap), and then blocked with 5% BSA in PBS/Sap at RT for 1 h. The cells were stained with rabbit anti-p85 antibody (1∶50, Cell Signaling Technology) in 1% BSA in PBS/Sap at RT for 2 h, followed by staining with AlexaFluor-594 goat anti-rabbit antibody (Life Technologies) in 1% BSA in PBS/Sap at RT for 1 h.

For detection of ectopically expressed FLAG-tagged GRP78 on the cell surface, HeLa cells were seeded at 5×10^3^ per well in 8-well chamber slides 24 h prior to transfection. Transfection was performed using BioT. Forty-eight hours after transfection, non-permeabilized cells were fixed and blocked as described above and then incubated with mouse anti-FLAG antibody (Sigma-Aldrich) in 1% BSA/PBS at 4°C for 1 h followed by incubation with AlexaFluor-488 goat anti-mouse antibody (Life Technologies) in 1% BSA/PBS at 4°C for 30 min. For detection of phosphatidylinositol 3,4,5-triphosphate (PI(3,4,5)P3), the cells were then treated with M.O.M.™ Mouse Ig Blocking Reagent (Vector Laboratories, Burlingame, CA) at RT for 1 h to block mouse immunoglobulin from primary mouse anti-FLAG antibody. The cells were subsequently permeabilized as described above and intracellular PI(3,4,5)P3 staining was performed as described [35]. For detection of p85 in HeLa cells, the cells were permeabilized and stained with the primary antibody at 37°C for 1 h and the secondary antibody at 37°C for 30 min.

All immunostained cells were mounted with Vectashield anti-fade medium containing DAPI (Vector Laboratories), and analyzed by a Zeiss LSM510 confocal microscope equipped with LSM 510 Version 4.2 SP1 acquisition software (Carl Zeiss). Representative images were taken with an EC Plan-Neofluar 40×/1.30 oil objective or a Plan-Apochromat 100×/1.4 oil DIC objective. Z-stack images were taken with a Plan-Apochromat 100×/1.4 oil DIC objective.

### Cell surface protein biotinylation and isolation

The cell surface proteins were biotinylated with non-permeable biotin, lysed in RIPA buffer and purified by NeutrAvidin agarose beads as previously described [32].

### Co-immunoprecipitation assay

The transfected cells were biotinylated and lysed with immunoprecipitation (IP) buffer (25 mM Tris-Cl, pH 7.4, 150 mM NaCl, 1% NP40). The cell lysates were subjected to monomeric avidin column (Thermo Scientific) and eluted with 2 mM D-biotin in PBS following manufacturer's instructions. The eluate was concentrated with Vivaspin 6 concentrators (10 kDa MWCO, Sartorius Stedim Biotech, Concord, CA), precleared with 50 µl Dynabeads protein G (Life Technologies) and subjected to incubation with 1 µg mouse anti-FLAG antibody (Sigma-Aldrich) or normal mouse IgG overnight at 4°C, followed by incubation with 50 µl Dynabeads protein G for 1 h at 4°C. After washing, the beads were boiled for 5 min in 2× SDS sample buffer, the samples were subjected to 10% SDS-PAGE electrophoresis and Western blot.

### Fluorescence-activated cell sorting (FACS) assay

The cells were detached with non-enzymatic cell dissociation solution (Sigma-Aldrich) at 37°C for 15 min and aliquoted to 1×10^6^ cells/tube, and incubated with 10% normal human serum in PBS for 20 min on ice to block Fc receptors on the cell surface. The cells were incubated with a saturating amount of mouse anti-GRP78 antibody (MAb159) (1 µg) for 40 min on ice in 100 µl of staining buffer (Dulbecco's PBS, 2% heat-inactivated fetal calf serum, 0.09% sodium azide), followed by AlexaFluor-488-conjugated goat anti-mouse secondary antibody (0.5 µg, Life Technologies) and suspended in ice-cold PBS containing 4,6-diamidino-2-phenylindole (DAPI, 1 µg/ml, Sigma-Aldrich) and subjected to FACS. The data were acquired by LSR II flow cytometer (Becton Dickinson, San Jose, CA) and analyzed using FlowJo software.

## Results

### Active promotion of cell surface GRP78 expression in cancer cells resistant to hormonal therapy

**Figure 1:**
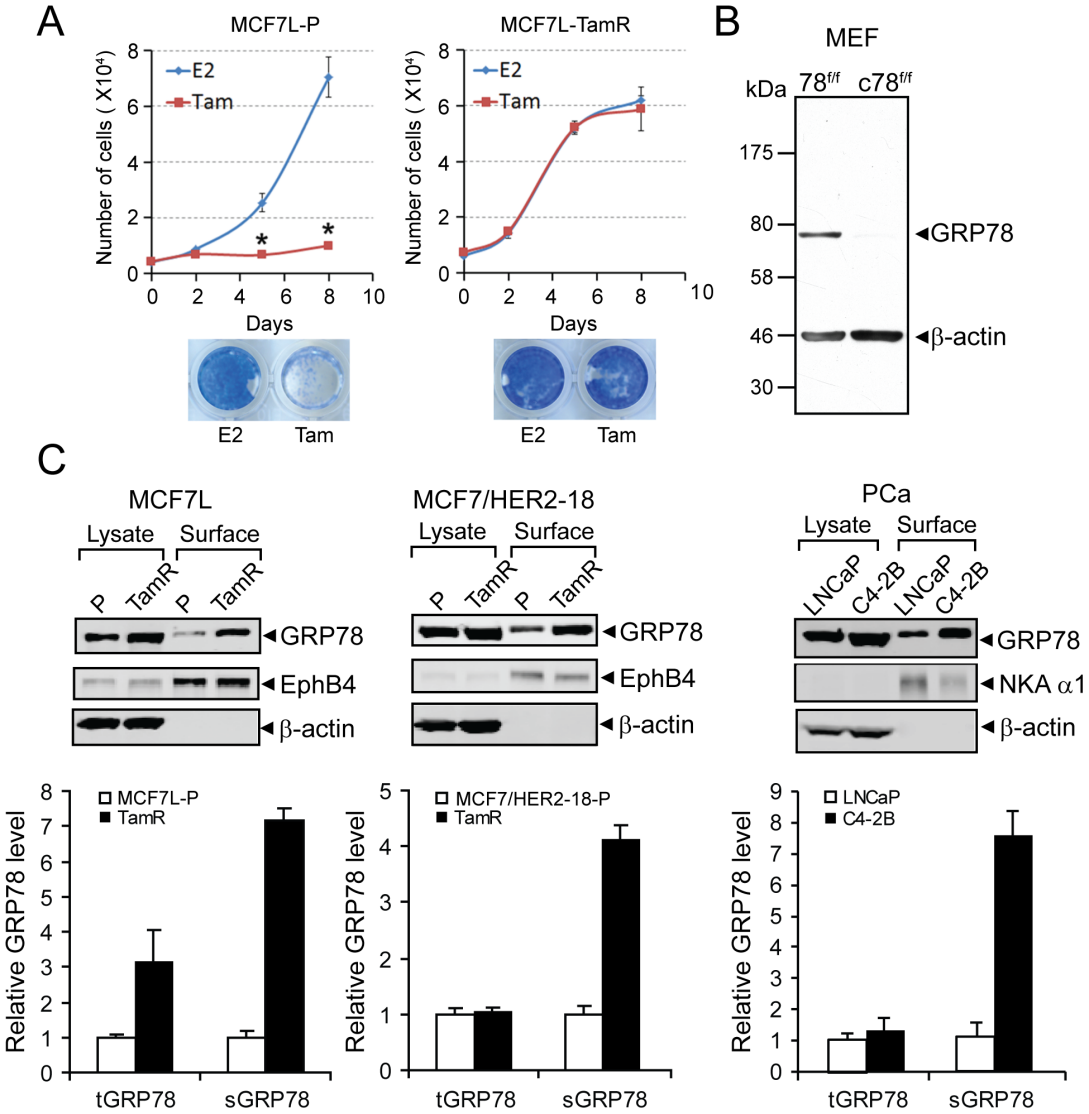
Active promotion of GRP78 to the cell surface in cancer cells resistant to therapy. (A) Validation of Tam resistant phenotype of the MCF7L-TamR cells. MCF7L-parental (P) and -TamR cells were pre-starved in phenol-red free (PRF) medium containing 5% CS-FBS for 5 d before subjected to estrogen (E2) (1 nM) or Tam (100 nM) treatment for 8 d. Cells were seeded in quadruplicates in 96-well plate and cell numbers were counted by an in situ cell cytometer. At day 8, cells were stained by methylene blue as shown in the lower panel. Error bars represent standard deviation; *P<0.001, two-sided t test, cell growth under Tam vs. E2. (B) Immunoblots of MEF cells using MAb159, with β-actin as loading control. Left lane showed MEF lysates from *Grp78 floxed/floxed* mice. Right lane showed abolishment of the GRP78 band after infection with adenovirus expressing the Cre-recombinase to knockout the *Grp78 floxed/floxed* alleles. (C) Representative Western blots for enhanced sGRP78 level in resistant cancer cells. Parental (P) and TamR derivatives of the human breast cancer cell models of MCF7L and MCF7/HER2-18, as well as the parental androgen sensitive LNCaP cell line and the androgen-independent C4-2B cells were subjected to biotinylation and NeutrAvidin agarose pull-down to enrich for cell surface protein. Cell surface GRP78 (sGRP78) and total intracellular GRP78 (tGRP78) in the cell lysate were probed by Western blot. The amount of total lysate was 10% of the amount used for the avidin pull-down. β-actin served as loading control for tGRP78, while membrane protein, EphB4, or Na, K-ATPase α1 (NKA α1) served as loading control of cell surface proteins in breast or prostate cancer cells (PCa), respectively. The experiments were repeated twice. The protein bands were quantitated and the relative levels of tGRP78 in the parental and resistant cell lines were normalized against β-actin, and sGRP78 level are normalized against EphB4 or NKA α1, respectively, which are shown by mean ± standard deviation (S.D.) in the graph below. The levels in parental cell lines and in androgen sensitive cell line, LNCaP are set as 1.

To test whether development of resistance to therapeutic treatment alters the expression of sGRP78 in cancer cells, we utilized three sets of cancer cell lines and their derivatives that have acquired resistance to the treatment regimen. The human breast cancer cell line MCF7L parental line (MCF7L-P) and a derivative tamoxifen resistant line (MCF7L-TamR) were generated by long term culture of MCF7L-P cells in PRF-medium containing 10% CS-FBS and 100 nM tamoxifen (Tam) until cell growth resumed (after 4 mo). In contrast to the MCF7L-P cells which grew in medium containing E2 but not in medium containing Tam, MCF7L-TamR cells showed equivalent growth rates when subjected to either estrogen (E2) or Tam (Figure 1A). The human breast cancer cell line MCF7/HER2-18-P which is an HER2-overexpressing clone and its Tam-R derivative was isolated after long term exposure to Tam for about 11 mo. The third pair of cells is the well-established androgen-sensitive human prostate adenocarcinoma cells, LNCaP, and its derivative, androgen-independent cell line, C4-2B.

For detection of GRP78, we utilized a high affinity monoclonal antibody MAb159 directed against human GRP78 that recognized only the GRP78 protein band (Figure 1B). Upon knockout of the *Grp78* floxed alleles in the MEFs by infection with adenovirus expressing the Cre-recombinase, the GRP78 band was abolished, confirming that MAb159 specifically recognizes GRP78. For detection of sGRP78, the cells were biotinylated and the cell surface proteins were purified by NeutrAvidin agarose pull-down. From Western blot analysis of the biotinylated proteins and the total lysate, the levels of sGRP78 and total intracellular GRP78 were determined for each cell line, with EphB4 and NKA α1 serving as loading controls for cell surface proteins for breast cancer and prostate cancer cell lines, respectively. β-actin, a cytosolic protein, served as loading control for the total cell lysates. Representative Western blot analyses for detection of sGRP78 and total GRP78 are shown, with the GRP78 levels after quantitation and normalization to the respective loading controls graphed below and shown with standard deviations (Figure 1C). The absence of β-actin in the proteins pull-down by NeutrAvidin confirmed the lack of intracellular protein contamination in the cell surface protein fractions. For MCF7L-P cells which expressed a moderate basal level of GRP78, there was a 3-fold increase in total GRP78 but a 7-fold increase in sGRP78 in the TamR cells. For MCF7/HER2-18-P cells which expressed a higher basal level of GRP78, the TamR cells only showed minimal increase in total GRP78 but a 4-fold increase in sGRP78. For the androgen-independent C4-2B cells, the total GRP78 increase over the androgen-sensitive LNCaP cells was minor but there was about a 7-fold increase in sGRP78. Collectively, these results imply that the increase in sGRP78 in resistant cell lines does not simply parallel the increase in the total amount of GRP78, rather the development of resistance promotes special mechanism(s) to enhance GRP78 localization to the cell surface.

### ER stress further elevates cell surface GRP78 level in resistant cancer cells

**Figure 2:**
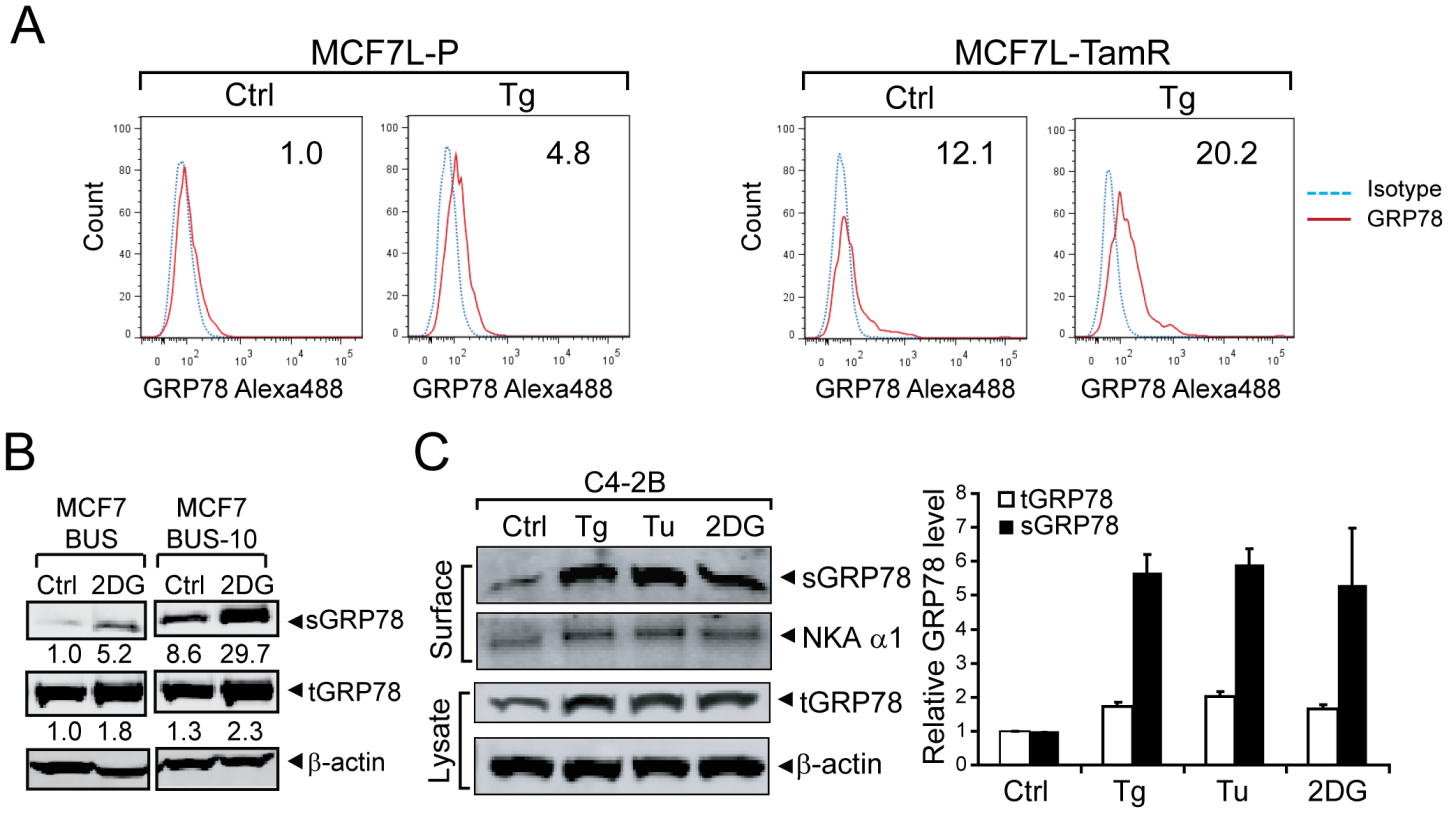
ER stress further elevates sGRP78 expression level in cancer cells. (A) Parental and tamoxifen-resistant MCF7L cells were either untreated (Ctrl) or treated with 300 nM thapsigargin (Tg) for 16 h. Cell surface GRP78 were measured by FACS. Representative FACS profiles are shown and percentages of positive cells are indicated on the upper right corner. Blue dashed line, isotype control; red solid line, anti-GRP78 Ab. (B) Estrogen starvation sensitive human breast cancer cell line, MCF7/BUS, and its resistant derivative clone, MCF7/BUS-10, were either untreated (Ctrl) or treated with 10 mM 2-deoxyglucose (2DG) for 24 h. The cells were biotinylated and cell surface proteins purified by NeutrAvidin agarose pull-down. sGRP78 and tGRP78 were detected by Western Blot. β-actin served as loading control. The fold changes in sGRP78 and tGRP78 are shown below and the control condition in MCF7/BUS cells was set as 1. (C) Same as (B) except C4-2B cells treated with Tg, Tu (tunicamycin) or 2DG. NKA α1 served as cell surface protein loading control. The relative levels of tGRP78 and sGRP78 are normalized against β-actin or NKA α1, respectively, and expressed as the mean ± S.D. from two independent experiments in graph (right).

Previously we reported that Tg, which inhibits the ER ATPase causing Ca^2+^ efflux from the ER, promotes translocation of GRP78 from the ER to the cell surface in human embryonic kidney 293T cells [32]. However, whether this applies to cancer cell lines which already exhibit moderate to high levels of GRP78 is not known. As cancer cells are routinely exposed to ER stress in the tumor microenvironment, it is important to determine whether cancer cells that have developed resistance to therapy are capable of further elevating sGRP78 expression in response to ER stress. To test these, we treated resistant cells with different inducers of ER stress and measured sGRP78 by FACS as well as by biochemical approaches. In the first approach, the MCF7L-P and TamR cell lines were treated with Tg. Cell surface GRP78 was detected by FACS analysis (Figure 2A). For the MCF7L-P cell line, treatment with Tg led to a 4.8-fold increase in sGRP78. For the MCF7L-TamR cell line which already exhibits 12-fold higher level of sGRP78 compared to the parental MCF7L-P cells, Tg treatment further elevates sGRP78 expression to 20-fold (Figure 2A).

In the second approach, we utilized the estrogen-dependent cell line MCF-7/BUS and its derivative MCF-7/BUS-10 cell line which has acquired resistance to estrogen starvation [43]. The cells were biotinylated and cell surface proteins subjected to NeutrAvidin agarose beads pull-down. The levels of sGRP78 and total intracellular GRP78 were determined by Western Blot (Figure 2B). In agreement with MCF7L and the MCF7/HER2-18 cell lines, development of resistance in the MCF-7/BUS-10 model substantially increased the amount of sGRP78 (by 9-fold), while the total intracellular amount was moderately increased (by 1.3-fold). For both the parental and resistant cell lines, treatment of cells with 2DG, an inducer of ER stress via inhibition of glycolysis and glycosylation, further elevated sGRP78 expression by about 5.2-fold in the parental line and 3.5-fold in the resistant line (Figure 2B).

We next examined the level of sGRP78 and total GRP78 in the androgen-independent prostate cancer cell line C4-2B, with or without ER stress (Figure 2C). In these studies, in addition to Tg and 2DG treatment, we added another ER stress inducer, tunicamycin (Tu), which creates ER stress by blocking N-linked protein glycosylation, thereby causing the accumulation of underglycosylated proteins in the ER. In the Western blot analysis, β-actin and NKA α1 served as loading controls for total and cell surface proteins, respectively. As in the case for the resistant breast cancer cells, ER stress caused a moderate increase of total GRP78 (by about 2-fold) as these cells already expressed high basal level of GRP78 compared to their sensitive parental cells. Under all three ER stress conditions, sGRP78 level increased by 5- to 6-fold, compared to non-stressed cells. Collectively, these results show that diverse ER stress stimuli are capable of actively promoting sGRP78 expression in both drug sensitive and resistant cancer cells, and that development of therapeutic resistance in combination with ER stress further enhances sGRP78 expression.

### Cell surface GRP78 forms complex with the PI3K

**Figure 3:**
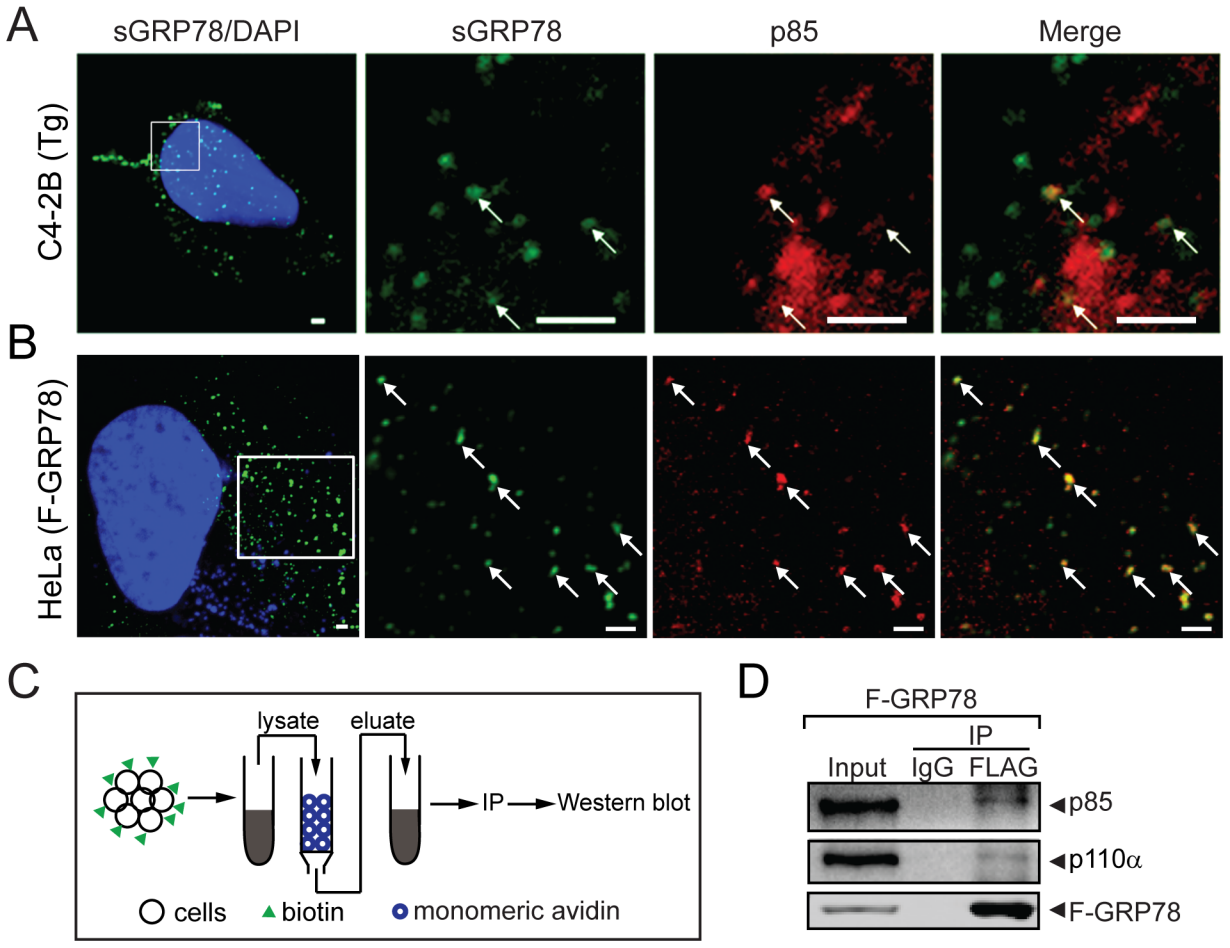
Cell surface GRP78 forms complex with the PI3K. (A) Confocal microscopy detection of co-localization of endogenous sGRP78 with p85 in C4-2B cells treated with Tg for 8 h. Non-permeabilized cells were first stained with anti-GRP78 antibody (MAb159) to detect sGRP78 (*green*) (left). The cells were then permeabilized and then stained with anti-p85 antibody (*red*). (B) Co-localization of cell surface F-GRP78 with p85 in HeLa cells. Non-permeabilized HeLa cells transfected with the F-GRP78 expression vector were first stained with anti-FLAG antibody to detect surface F-GRP78 (*green*) (left), then permeabilized and stained with anti-p85 antibody (*red*). In both (A) and (B), the nuclei were stained by DAPI (*blue*) and the boxed regions (white square) of the compressed Z-stack images (left panels) were enlarged and shown on the right panels in single confocal section planes (thickness 0.38 µm).The merged images showed co-localizations (*yellow*) of cell surface GRP78 and p85 detected at multiple corresponding sites (*white arrows*). Scale bars represent 2 µm. (C) Scheme for detection of interaction between cell surface F-GRP78 and p85. Biotinylated cell surface proteins were purified by monomeric avidin column, followed by immunoprecipitation (IP) and Western blot. (D) 293T cells were transfected with F-GRP78 expression vector. Cell surface proteins eluted from the monomeric avidin column (input) as described in (C) were subjected to immunoprecipitation (IP) with either anti-FLAG antibody or isotype IgG serving as negative control. F-GRP78, p85 and p110α levels in immunoprecipitated complex were measured by Western blot with anti-FLAG, anti-p85 and anti-p110α antibodies.

Recent studies suggest that perturbation of sGRP78 alters the PI3K/AKT pathway, however, how that is achieved is not well understood [14], [16]. To address this, we determined whether sGRP78 co-localized with PI3K at the cell surface. C4-2B, with high endogenous sGRP78 and relative ease of culture, presents a suitable cell model system. To maximize sGRP78 expression, the cells were treated with Tg. In non-permeabilized cells, antibody staining will primarily detect GRP78 on the cell surface, rather than the highly abundant intracellular GRP78, which localizes majorly in the perinuclear ER region. Following 8 h of Tg treatment, endogenous sGRP78, as visualized by immunofluorescence staining with the MAb159 monoclonal antibody, was detected scattered over the cell surface of C4-2B cells (Figure 3A). To detect the PI3K regulatory subunit p85 which is intracellular, the MAb159 immunostained cells were permeabilized followed by staining with antibody against p85. As shown in representative images, co-localization of endogenous sGRP78 and p85 in multiple sites were observed for a subfraction of sGRP78 and p85 (Figure 3A, arrows).

To extend these observations, we transfected HeLa cells with the expression vector for FLAG-GRP78 (F-GRP78). The use of GRP78 marked with the FLAG epitope allowed the use of the highly specific anti-FLAG antibody to detect cell surface GRP78 expression in non-permeabilized cells. HeLa cells were used since they adhere more strongly to microscopic slides, which is critical for the multiple steps of experimental manipulations of the transfected cells. Our results showed abundant co-localization of cell surface F-GRP78 with p85 (Figure 3B).

Next, to determine biochemically whether sGRP78 formed complex with PI3K, 293T cells were transfected with F-GRP78. 293T cells were used due to their high transfection efficiency. The use of F-GRP78 allowed us to immunoprecipitate GRP78 with high specificity and affinity. The experimental design is shown in Figure 3C. After transfection, the cells were biotinylated and the cell lysates were incubated with monomeric avidin agarose beads which allow isolation of biotinylated cell surface proteins by mild elution condition due to its weak affinity to biotinylated protein. The eluted cell surface proteins were immunoprecipitated with anti-FLAG antibody or the isotype IgG control. The immunoprecipitates were then subjected to Western blot and probed with antibodies against p85 and p110α, as well as the anti-FLAG antibody. We observed that cell surface F-GRP78 formed complex with both the regulatory (p85) and catalytic (p110α) subunits of PI3K, in contrast to the IgG control which showed no binding (Figure 3D). Collectively, these results suggest that sGRP78 directly or indirectly interacts with p85 and p110α.

### Cell surface GRP78 overexpression stimulates PI(3,4,5)P3 production and co-localization

**Figure 4:**
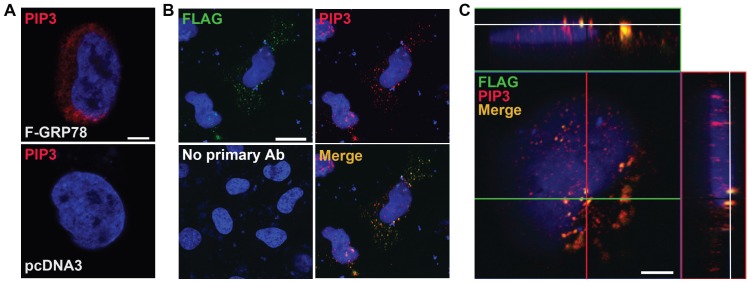
Overexpression of cell surface GRP78 stimulates PIP3 production and co-localization. (A) HeLa cells transfected with either F-GRP78 expression vector or pcDNA3 were permeabilized and immunostained with anti-PI(3,4,5)P3 antibody (*red*). Scale bar represents 5 µm. (B) Non-permeabilized HeLa cells expressing F-GRP78 were immunostained with anti-FLAG antibody (*green*) and then were permeabilized and immunostained with anti-PI(3,4,5)P3 antibody (*red*). The nuclei were stained by DAPI (*blue*). The cells were subjected to confocal microscopy. Representative images show co-localizations of FLAG-tagged sGRP78 and PIP3 (*yellow*). Scale bar represents 20 µm. (C) The section at the top layer of the cell from confocal Z-stack images shows co-localizations of FLAG-tagged sGRP78 and PIP3 on the cell surface. White lines indicate the section level. The green and red lines indicate the locations of the front and side views respectively. Scale bar represents 5 µm.

The complex formation between sGRP78 and PI3K suggests that it may lead to modulation of PI3K activity. Upon activation, PI3K localizes to the cell surface and phosphorylates PI(4,5)P2 leading to PI(3,4,5)P3 (referred to below as PIP3) production. To test the consequence of sGRP78 expression on PIP3 production, HeLa cells transfected with either the expression vector for F-GRP78 or the empty vector pcDNA3 were immunostained with anti-PIP3 antibody and examined by confocal microscopy. Representative images are shown in Figure 4A. Higher level of PIP3 was evident in cells expressing F-GRP78 compared to pcDNA3 transfected cells. To examine whether F-GRP78 on the cell surface co-localized with PIP3, the transfected cells were first stained with anti-FLAG antibody in the non-permeabilized setting to detect F-GRP78 on the cell surface, then the cells were permeabilized and stained with antibody against PIP3, which is intracellular. The merged image showed co-localization of sGRP78 and PIP3 in multiple sites on the cell surface (Figure 4B). Analysis of the section of confocal Z-stack images at the top layer of the cell further illustrated the co-localization of F-GRP78 and PIP3 on the cell surface (Figure 4C). The front and side views of section planes shown as the green and red lines, respectively, revealed co-localizations as demonstrated by co-staining of F-GRP78 and PIP3 on the cell surface.

### Insertion mutant of GRP78 at the N-terminal domain retains protein expression and ability to translocate to the cell surface

**Figure 5:**
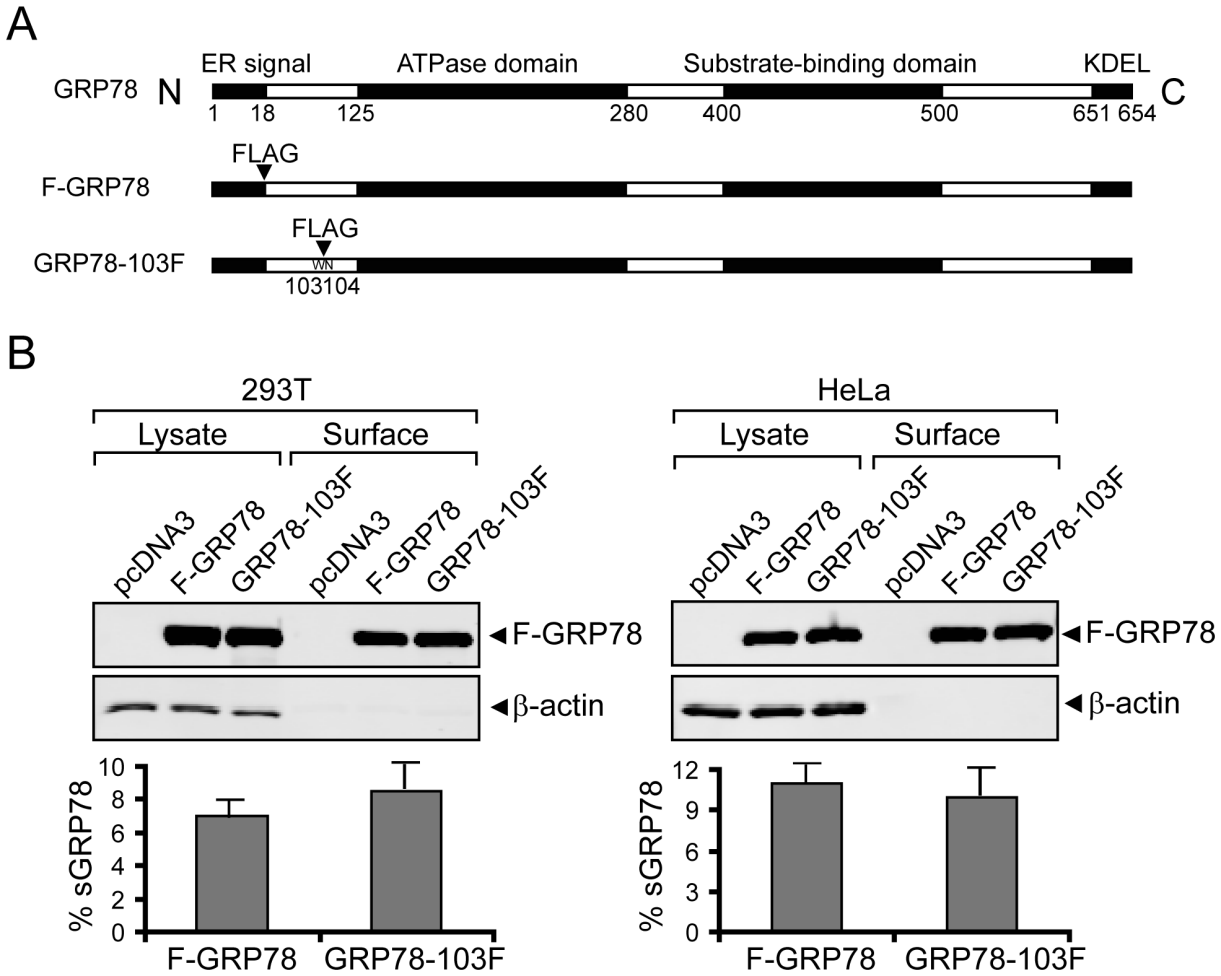
GRP78-103F mutant protein exhibits normal translocation to the cell surface. (A) Schematic diagram of human full length GRP78 with the FLAG tag either inserted immediately after ER signal peptide at the N-terminus of GRP78 (F-GRP78) or between amino acid W103-N104 (GRP78-103F). The locations of the ER signal peptide, ATPase domain, substrate-binding domain and KDEL motif are indicated. (B) Human HEK293T and HeLa cells transiently transfected with expression plasmids encoding F-GRP78 or GRP78-103F were biotinylated and subjected to NeutrAvidin agarose pull-down to isolate cell surface proteins. GRP78 expression levels in total lysate and cell surface were detected by Western blot. The amount of total lysate was 10% of the amount used for NeutrAvidin agarose pull-down. The band intensities were quantitated and the percentage of cell surface GRP78 for each construct was calculated and shown as the mean ± S.D. from three independent experiments in the graph below.

It has been reported the N-terminal region of GRP78 around amino acids (aa) 98–115 binds external ligands and autoimmune antibody against GRP78 leading to AKT phosphorylation [36]. This suggests that this domain of GRP78 may be important for interaction with PI3K and PIP3 production. Thus, in designing a mutant to disrupt this region, we generated a new construct of GRP78, referred to below as GRP78-103F, by inserting a FLAG tag between aa 103 and 104 (Figure 5A). The FLAG-epitope allowed us to detect expression of this mutant protein. The position of the insertion was chosen since it is in-between the conserved domains of GRP78 deduced by sequence alignment map [44] and it lies outside of the ATP binding domain of GRP78 located between aa 125 and aa 280. This will avoid disrupting the critical ATPase function of GRP78 as well as preserving the ER targeting signal at the N-terminus. The C-terminal half of GRP78 containing the substrate binding domain and the KDEL ER retention signal should also not be affected. Furthermore, since it does not disrupt conserved domains of GRP78 and is not related to known key aa positions required for maintaining correct conformation and function of ATPase domains from published data, it is predicted not to affect the structural integrity of the GRP78 protein.

Prior to analyzing its ability to interact with PI3K, we compared the stability of GRP78-103F and its ability to translocate to the cell surface with wild-type GRP78 in two human cell lines. Thus, 293T and HeLa cells were transiently transfected with equivalent amounts of expression vector for either F-GRP78 WT or GRP78-103F. The cells were biotinylated and total sGRP78 was measured biochemically as described above. From analysis of the total cell lysate, it is evident that the expression levels of intracellular F-GRP78 and GRP78-103F were similar, suggesting that insertion mutation does not affect protein expression (Figure 5B). From the analysis of cell surface protein, the level of cell surface F-GRP78 and GRP78-103F in 293T and HeLa cells were also very similar (Figure 5B). These results indicate that the insertion mutant GRP78-103F is able to maintain protein expression level and translocation to the cell surface as the wild-type protein.

### Insertion mutation at GRP78 N-terminal domain suppresses p85 complex formation and PIP3 production

**Figure 6:**
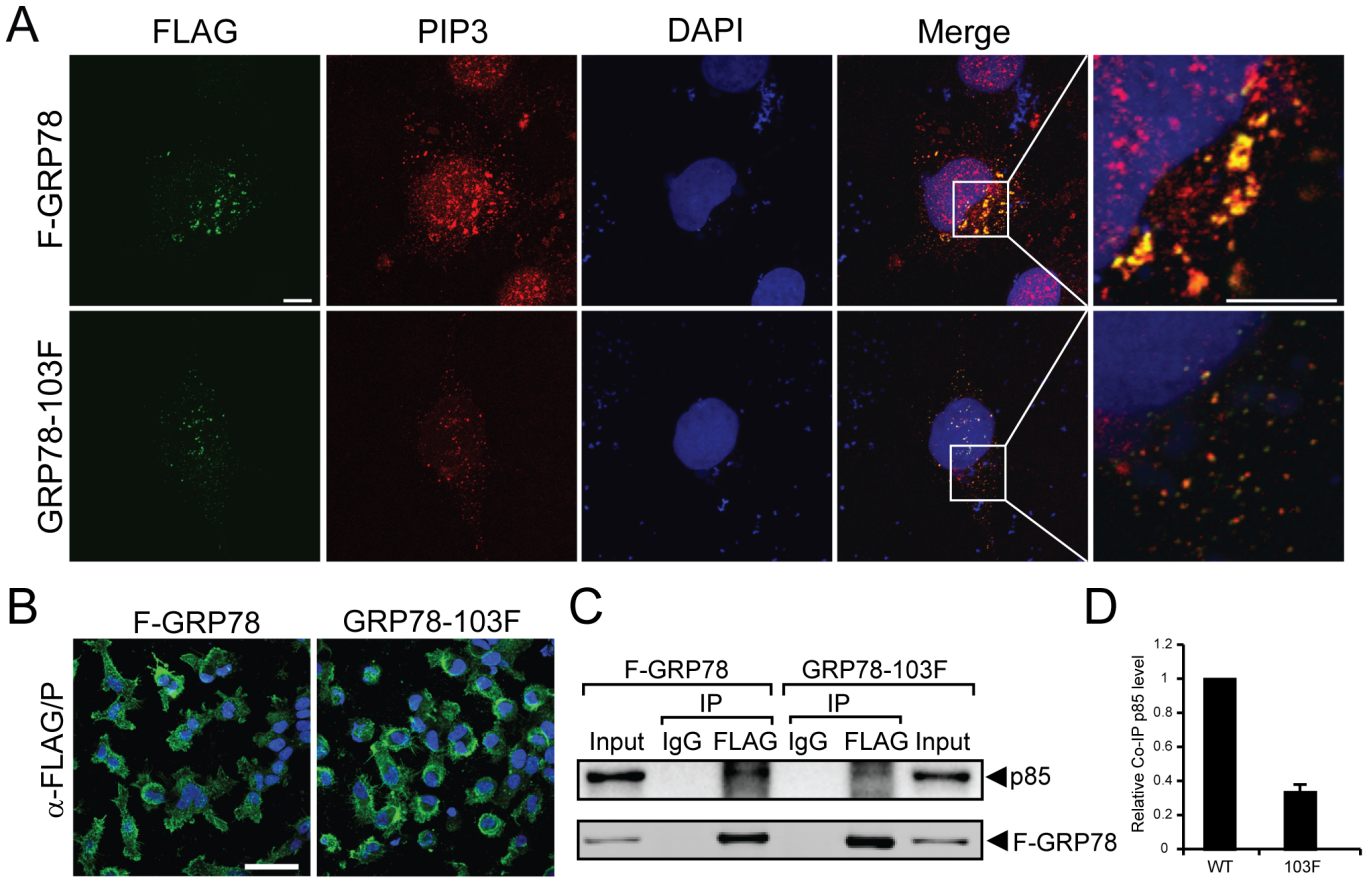
GRP78-103F mutant protein suppresses p85 complex formation and PIP3 production. (A) HeLa cells were transfected with either F-GRP78 or GRP78-103F expression vectors. Non-permeabilized cells were immunostained with anti-FLAG antibody (*green*) and then immunostained with anti-PIP3 antibody (*red*) following permeabilization. The nuclei were stained by DAPI (*blue*). Representative compressed confocal Z-stack images are shown. The merged images showed co-localization of F-GRP78 with PIP3 (*yellow*). The boxed areas in the merged images were enlarged and shown as single confocal section planes (thickness 0.38 µm). Scale bars represent 10 µm. (B) Intracellular expression of F-GRP78 and GRP78-103F was assessed by immunostaining of permeabilized cells with anti-FLAG antibody. (C) Co-immunoprecipitation (Co-IP) of ectopically expressed sGRP78 and p85 in 293T cells. 293T cells were co-transfected with F-GRP78 or GRP78-103F and p85 for 48 h. Cell surface proteins eluted from the monomeric avidin column from lysates of biotinylated cells (input) as described in Figure 3C were subjected to immunoprecipitation with either anti-FLAG antibody or IgG isotype control, followed by Western blot with anti-p85 and anti-FLAG antibody to detect complex formation. (D) The protein bands in the (C) panel were quantitated and the relative co-immunoprecipitated p85 levels normalized against immunoprecipitated F-GRP78 (WT) or GRP78-103F (103F) are plotted in the graph. The levels are shown as the mean ± S.D. from two independent experiments, with the level in WT set as 1.

Next we compared the ability of F-GRP78 and GRP78-103F to promote PIP3 production using confocal microscopy. HeLa cells transfected with F-GRP78 showed staining of F-GRP78 on the cell surface in non-permeabilized cells, associating with robust stimulation of PIP3 production. Merged images further showed abundant co-localization of cell surface F-GRP78 with PIP3 (Figure 6A). Surprisingly, despite similar expression level at the cell surface as F-GRP78 as revealed by protein biotinylation (Figure 5B), FLAG staining of sGRP78-103F in non-permeabilized cells was reduced by about 50% compared to F-GRP78 (Figure 6A). By staining the permeabilized cells with immunofluorescent anti-FLAG antibody, we further determined that the transfection efficiency into HeLa cells was high and the expression level for both proteins was equivalent (Figure 6B). This suggests that while GRP78-103F is stably expressed and can translocate to the cell surface, the FLAG-epitope in GRP78-103F was less accessible to staining on the cell surface in the non-permeabilized cells. The level of PIP3 production was much reduced in the GRP78-103F cells compared to F-GRP78 (Figure 6A). The merged images showed reduced co-localization of GRP78-103F with PIP3.

To determine whether the mutation affects complex formation with PI3K, we co-transfected the expression vectors for either F-GRP78 or GRP78-103F with p85 in 293T cells. After transfection, the cells were biotinylated and the cell lysates were incubated with monomeric avidin agarose beads. The eluted cell surface proteins were subjected to immunoprecipitation with anti-FLAG antibody or the isotype IgG control, followed by Western blot for detection of p85 and F-GRP78 levels in the immunoprecipitates (Figure 6C). The protein band intensities were quantitated and the relative levels of p85 in the co-immunoprecipitate normalized against immunoprecipitated F-GRP78 or GRP78-103F in the complex were determined and graphed (Figure 6D). Our results showed reduced p85 complex formation with GRP78-103F, compared with F-GRP78 (Figure 6C,D). Collectively, these results suggest that insertion mutation at the N-terminal domain of GRP78 disrupts the sGRP78 and p85 complex and suppresses its ability to stimulate PIP3 production.

## Discussion

Overexpression of GRP78 has been documented in a wide range of human tumors and confers resistance to hormonal as well as cytotoxic therapy. The discovery that GRP78 can also localize to the cell surface under pathophysiologic conditions such as cancer, opens up new mechanisms whereby this protein may exert its pro-proliferative and anti-apoptotic function in cancer. Cellular proteins such as MTJ-1 and Par-4 have been reported to facilitate trafficking of GRP78 from the ER to the cell surface [29], [45]; however, their association with sGRP78 is context dependent [46]. Our studies on sGRP78 in model cell systems and in cancer cell lines studies uncovered several novel observations.

Due to expression of sGRP78 in tumor but not normal cells in vivo, sGRP78 is recognized as a promising target for anti-cancer therapy. This can be achieved through direct disruption of sGRP78 function or a mediator for delivery of toxic reagents into cancer cells [4], [5], [15], [16], [21], [37], [47]. Here we compared directly the level of total and sGRP78 in four matched pairs of sensitive and resistant cancer cells. Our finding that development of resistance in all four sets of cancer cells leads to a much larger increase in sGRP78 than total GRP78 implies that as cancer cells acquire resistance to therapy, they also develop mechanism(s) to actively promote sGRP78 expression. Furthermore, most cancer cells are subjected to ER stress. In this study, we found that in both sensitive and resistant cancer cell lines, the level of sGRP78 can still be further elevated by ER stress. Previously, we observed that enforced expression of GRP78 can lead to sGRP78 expression independent of ER stress [32], and that additional ER stress can further elevate the level of sGRP78. It is also possible that the level of ER stress in the cancer cells is only moderate thus it can be further stressed to deliver more GRP78 to the cell surface. Irrespective of the mechanism, our results suggest that therapeutic agents targeting sGRP78 will be highly efficacious against resistant cancer cells, and even more so against resistant cells under ER stress, which is characteristic of residual cancer cells that survive therapy [48].

The PI3K/AKT pathway is a major pro-proliferative and pro-survival pathway in cancer and blockage of this pathway holds great promise for suppressing tumor progression as well as therapeutic resistance. We have recently shown that GRP78 is not only critically required for prostate tumorigenesis and leukemogenesis, it is also required for AKT activation both in vivo and in vitro [35], [49]. Additionally, GRP78 knockdown potently inhibited serum-induced production of PIP3, but not ERK or p38 MAPK activation. In cells where GRP78 is knockdown by siRNA, both intracellular and sGRP78 are downregulated [50], so the effect observed could be mediated by both or either form of GRP78. Here using imaging and biochemical approaches, we provide the proof-of-principle that sGRP78 forms complex with the subunits of PI3K. While whether the interaction between sGRP78 and PI3K is direct or indirect awaits further investigation, the interaction is selective since a recent study showed that sGRP78 did not form complex with another cell surface protein EphB2 [37]. Stress induction of sGRP78 and their co-localization with p85 may be transient, thus future studies examining the interaction between sGRP78 and PI3K at the kinetic level in different cell types and under different conditions will provide more information on the dynamics of this interaction. Overexpression of sGRP78 leads to an increase in PIP3 production and co-localization of sGRP78 with PIP3. Additionally, insertion mutation of GRP78 at the junction of aa 103 and aa 104 resulted in reduced complex formation with p85 and PIP3 production. Unfortunately, our attempts to establish stable expression of internal deletion mutants at the N-terminal regions were unsuccessful due to rapid degradation of the mutant proteins as previously observed ([16], data not shown). While this requires further investigation, our mutational studies provide new evidence that the N-terminal domain of GRP78 is critical for its ability to stimulate PI3K activity, which is in agreement with recent studies that the N-terminus domain of GRP78 is important for oncogenic PI3K signaling [25], [36]. These findings provide a potential mechanistic explanation on how sGRP78 can serve as regulator of the PI3K pathway and expand the function of GRP78 beyond the role of molecular chaperone and ER stress signaling regulator.
